# Supplementary material for: Peripheral blood transcriptome identifies high-risk benign and malignant breast lesions
Source: PLoS One. 2020 Jun 4;15(6):e0233713. doi: 10.1371/journal.pone.0233713 (PMC7272048; doi:10.1371/journal.pone.0233713)
Supplement: S2 Checklist — (DOCX) [file pone.0233713.s002.docx]

STROBE Statement—checklist of items that should be included in reports of observational studies

|  | Item No. | Recommendation | Page  No. | Relevant text from manuscript |
| --- | --- | --- | --- | --- |
| **Title and abstract** | 1 | (*a*) Indicate the study’s design with a commonly used term in the title or the abstract | P2 | We utilize this technique in a case-control study |
|  |  | (*b*) Provide in the abstract an informative and balanced summary of what was done and what was found | P2 | We utilize this technique in a case-control study to identify candidate transcriptomic biomarkers able to differentiate women with breast lesions from normal controls. Ten gene biomarkers were identified (*YWHAQ, BCLAF1, WSB1, PBX2, DDIT4, LUC7L3, FKBP1A, APP, HERC2P2, FAM126B*). |
| Introduction | | | |  |
| Background/rationale | 2 | Explain the scientific background and rationale for the investigation being reported | P4-5 | Breast lesions at an early stage are usually asymptomatic and undetectable by self-examination, resulting in delayed treatment… Thus novel, minimally invasive biomarkers have been sought, to improve the early detection of breast lesions. |
| Objectives | 3 | State specific objectives, including any prespecified hypotheses | P5 | In the present study, we compare the blood gene expression profiles in women with breast lesions and control women with no breast disease in order potentially to develop a non-invasive test for early stage breast cancer and breast lesions. The transcriptomic biomarkers of breast lesions were identified and the roles of these genes in biological processes and functions were analyzed for clues to the pathogenesis of breast lesions. |
| Methods | | | |  |
| Study design | 4 | Present key elements of study design early in the paper |  | N/A |
| Setting | 5 | Describe the setting, locations, and relevant dates, including periods of recruitment, exposure, follow-up, and data collection | P5-6 | This study was approved by the Ethics Committee of the Qingdao Central (Tumor) Hospital (IRB no. KY-P201803601) on January 30^th^ 2019. Participants were recruited to this study from January 31^st^ 2019 to June 30^th^ 2019. Sample acquisition was conducted between January 31^st^ 2019 and June 30^th^ 2019 at the Qingdao Central (Tumor) Hospital. 151 participants were enrolled, including 44 healthy controls and 107 patients with breast lesions (50 high risk lesions and 57 breast cancer). Written informed consent was obtained from all study participants and approved by Ethics Committee of Qingdao Central (Tumor) Hospital. All authors in this manuscript had access to individual participants’ information and medical records, and data was scrubbed after information collection. |
| Participants | 6 | (*a*) *Cohort study*—Give the eligibility criteria, and the sources and methods of selection of participants. Describe methods of follow-up  *Case-control study*—Give the eligibility criteria, and the sources and methods of case ascertainment and control selection. Give the rationale for the choice of cases and controls  *Cross-sectional study*—Give the eligibility criteria, and the sources and methods of selection of participants | P6 | 151 participants were enrolled, including 44 controls and 107 patients with breast lesions (50 high risk lesions and 57 breast cancer). A total of 107 blood samples from patients with breast lesions was obtained. The study population comprised 107 female adult patients (age range, 23-78 years; mean age: 50.6 ± 11.2 years), including 50 women with high-risk breast lesions and 57 breast cancer patients. All patients were recruited before they had undergone any form of treatment, including endocrinotherapy, radio/chemo-therapy, targeted therapy or surgery. The breast lesion cohorts were categorized according to pathological examination. All patients underwent mammography or ultrasound, and the results were analyzed and categorized according to the Breast Imaging Reporting and Data System (BI-RADS) Grades. In cases where the grades of mammography and ultrasound were inconsistent, the higher grade was adopted. High-risk lesions were defined as BI-RADS Grades 3 to 5 with no evidence of cancer at biopsy. |
|  |  | (*b*) *Cohort study*—For matched studies, give matching criteria and number of exposed and unexposed  *Case-control study*—For matched studies, give matching criteria and the number of controls per case |  | N/A |
| Variables | 7 | Clearly define all outcomes, exposures, predictors, potential confounders, and effect modifiers. Give diagnostic criteria, if applicable |  | N/A |
| Data sources/ measurement | 8* | For each variable of interest, give sources of data and details of methods of assessment (measurement). Describe comparability of assessment methods if there is more than one group | P8 | To accelerate the screening of breast lesion-specific gene expression signatures, an ensemble learning strategy called AdaBoost was executed. Instead of making restrictive assumptions regarding the training set as in traditional data mining methods, this boosting method first creates a set of weak classifiers by assigning them appropriate extra weights and then combines these weak classifiers into a strong classifier. AdaBoost has important and significant advantages in both accuracy and training time as compared with other data mining methods. The transcriptomic features of the breast lesions were identified and used to construct the predictive model by AdaBoost. To classify the breast lesion group and the normal control group, the area under the receiver operating characteristic curve (AUC) sensitivity, specificity and accuracy were estimated in both the training and the test groups. |
| Bias | 9 | Describe any efforts to address potential sources of bias | P8 | To accelerate the screening of breast lesion-specific gene expression signatures, an ensemble learning strategy called AdaBoost was executed. Instead of making restrictive assumptions regarding the training set as in traditional data mining methods, this boosting method first creates a set of weak classifiers by assigning them appropriate extra weights and then combines these weak classifiers into a strong classifier. AdaBoost has important and significant advantages in both accuracy and training time as compared with other data mining methods. |
| Study size | 10 | Explain how the study size was arrived at |  | N/A |

Continued on next page

| Quantitative variables | 11 | Explain how quantitative variables were handled in the analyses. If applicable, describe which groupings were chosen and why |  | N/A |
| --- | --- | --- | --- | --- |
| Statistical methods | 12 | (*a*) Describe all statistical methods, including those used to control for confounding | P8-9 | **Microarray data analysis**  To accelerate the screening of breast lesion-specific gene expression signatures, an ensemble learning strategy called AdaBoost was executed. Instead of making restrictive assumptions regarding the training set as in traditional data mining methods, this boosting method first creates a set of weak classifiers by assigning them appropriate extra weights and then combines these weak classifiers into a strong classifier. AdaBoost has important and significant advantages in both accuracy and training time as compared with other data mining methods [23]. The transcriptomic features of the breast lesions were identified and used to construct the predictive model by AdaBoost. To classify the breast lesion group and the normal control group, the area under the receiver operating characteristic curve (AUC) sensitivity, specificity and accuracy were estimated in both the training and the test groups.  **Bioinformatics analysis**  The GO and KEGG annotations of the selected transcriptomic genes were queried from the COXPRESdb v7 database [24] . The protein-protein interactions between each transcriptomic feature and its first neighbouring protein counterpart with number less than 20 were downloaded from the STRING database with a total confidence greater than or equal to 0.7. Gene-annotation enrichment analysis using the cluster Profiler R package was performed on signature genes and their correlative proteins. Gene Ontology (GO) terms were identified with a strict cutoff of adjusted p < 0.05 corrected with the Benjamini–Hochberg （BH） method and a false discovery rate (FDR) of less than 0.05. Reactome pathways were also identified, with a strict cutoff of p < 0.05 corrected with the BH method and a false discovery rate (FDR) of less than 0.05. The protein-protein interaction network and gene network with the final biomarkers was carried out with Cytoscape software. |
|  |  | (*b*) Describe any methods used to examine subgroups and interactions |  | N/A |
|  |  | (*c*) Explain how missing data were addressed |  | N/A |
|  |  | (*d*) *Cohort study*—If applicable, explain how loss to follow-up was addressed  *Case-control study*—If applicable, explain how matching of cases and controls was addressed  *Cross-sectional study*—If applicable, describe analytical methods taking account of sampling strategy |  | N/A |
|  |  | (*e*) Describe any sensitivity analyses |  | N/A |
| Results | | | | |
| Participants | 13* | (a) Report numbers of individuals at each stage of study—eg numbers potentially eligible, examined for eligibility, confirmed eligible, included in the study, completing follow-up, and analysed | P9 | For this study a total of 151 blood samples was collected, including 44 controls and 107 breast lesions (50 high-risk breast lesions and 57 breast cancer lesions). Patients with breast cancer were older than the controls and older than those with high-risk lesions. Most subjects in the control group were aged less than 60 years, whereas about half (49/107) of the patients in the breast lesion cohort were older than age 60 (Table 1). The BI-RADS Grades of patients in the breast lesion group are also summarized: for high-risk lesions, the number of lesions Grade 3 and 4 was similar; for breast cancer lesions, most of the patients were Grade 5 (Table 1). |
|  |  | (b) Give reasons for non-participation at each stage |  | N/A |
|  |  | (c) Consider use of a flow diagram |  | N/A |
| Descriptive data | 14* | (a) Give characteristics of study participants (eg demographic, clinical, social) and information on exposures and potential confounders | P10-11 | Table 1   \| **Table 1 The basic characteristics of Normal Control and Breast Lesions** \| \| \| \| \| --- \| --- \| --- \| --- \| \|  \| **Normal control** \| **Breast lesions** \| \| \| **High risk lesions** \| **Breast cancer** \| \| **Age(years)** \|  \|  \|  \| \| Min \| 26 \| 23 \| 33 \| \| Max \| 6 \| 68 \| 78 \| \| Mean \| 42.6±11.6 \| 44.9±10.0 \| 55.6±9.6 \| \|  \|  \|  \|  \| \| **Total-Age groups(years)** \|  \|  \|  \| \| 21-30 \| 8 \| 5 \| 0 \| \| 31-40 \| 12 \| 10 \| 4 \| \| 41-50 \| 12 \| 22 \| 14 \| \| 51-60 \| 11 \| 10 \| 19 \| \| 61-70 \| 0 \| 3 \| 17 \| \| 71-80 \| 1 \| 0 \| 3 \| \| Total \| 44 \| 50 \| 57 \| \|  \|  \|  \|  \| \| **BI-RADS Grades** \|  \|  \|  \| \| 3 \|  \| 23 \| 0 \| \| 4 \|  \| 27 \| 10 \| \| 5 \|  \| 0 \| 42 \| \| 6 \|  \| 0 \| 5 \| \| Total \|  \| 50 \| 57 \| |
|  |  | (b) Indicate number of participants with missing data for each variable of interest |  | N/A |
|  |  | (c) *Cohort study*—Summarise follow-up time (eg, average and total amount) |  | N/A |
| Outcome data | 15* | *Cohort study*—Report numbers of outcome events or summary measures over time |  | N/A |
|  |  | *Case-control study—*Report numbers in each exposure category, or summary measures of exposure | P14 | To construct the predictive model, we divided the total data into a training set and a test set in proportions of 7:3. The predictive model built on the training set that contained a total of 105 samples included 80 breast lesions and 25 normal controls. The performance of the predictive model was then evaluated by the completely independent samples in the test set, which contained a total of 46 samples, including 27 breast lesions and 19 normal controls. |
|  |  | *Cross-sectional study—*Report numbers of outcome events or summary measures |  | N/A |
| Main results | 16 | (*a*) Give unadjusted estimates and, if applicable, confounder-adjusted estimates and their precision (eg, 95% confidence interval). Make clear which confounders were adjusted for and why they were included |  | N/A |
|  |  | (*b*) Report category boundaries when continuous variables were categorized |  | N/A |
|  |  | (*c*) If relevant, consider translating estimates of relative risk into absolute risk for a meaningful time period |  | N/A |

| Other analyses | 17 | Report other analyses done—eg analyses of subgroups and interactions, and sensitivity analyses |  | N/A |
| --- | --- | --- | --- | --- |
| Discussion | | | | |
| Key results | 18 | Summarise key results with reference to study objectives | P16 | In this study we report a method for differentiating breast lesions -- including high-risk benign breast lesions and malignant breast lesions -- from normal controls using blood transcriptomic gene expression analysis. We collected blood samples from healthy control women with no breast disease and from breast lesion patients, and focused on identifying blood transcriptomic features that can distinguish the two groups. We identified ten genes that can detect breast lesions with an accuracy higher than 90%. These preliminary results are encouraging, but further research is needed for validation. |
| Limitations | 19 | Discuss limitations of the study, taking into account sources of potential bias or imprecision. Discuss both direction and magnitude of any potential bias | P21-22 | Our study has several limitations. First, the sample size was relatively small and different genes or more genes that have better discriminatory power may be validated among a larger independent cohort of patients. Second, the nature of the mechanisms driving the different transcriptomic biomarkers in peripheral blood is not yet clear, and the function of some biomarkers requires further study. We are currently exploring the expression differences of the ten candidate biomarkers between high-risk breast lesions and breast cancer, which study may be helpful for the differential diagnosis of high risk lesions and breast cancer. |
| Interpretation | 20 | Give a cautious overall interpretation of results considering objectives, limitations, multiplicity of analyses, results from similar studies, and other relevant evidence | P22 | Using peripheral blood gene expression profiles we identified ten transcriptomic biomarkers that could distinguish women with high-risk breast lesions and breast cancer from normal controls. Our model, based in the ten transcriptomic biomarkers identified, has shown good discriminatory power between breast lesion and control subjects. Our functional enrichment analysis suggested that our candidate biomarkers were mainly involved in apoptosis, TGF-beta signaling, adaptive immune system regulation, gene transcription and post-transcriptional protein modification. This study has therefore established a promising methodology for the non-invasive detection of breast lesions, and we have also shed light on the pathogenic mechanisms of breast cancer and provided clues to new targets for breast cancer therapy, especially therapies related to immune treatment. |
| Generalisability | 21 | Discuss the generalisability (external validity) of the study results | P17-21 | Among the ten candidate biomarkers we identified (*YWHAQ, BCLAF1,WSB1, PBX2, DDIT4, LUC7L3, FKBP1A, APP,HERC2P2,FAM126B*), five genes (*DDIT4, APP, FKBP1A, PBX2, YWHAQ*) were upregulated in breast lesion patients as compared with normal controls ,and the other five genes were downregulated (*FAM126B, BCLAF1, WSB1, LUC7L33, HERC2P2*.) There were a total of 147 proteins interacting with the ten transcriptomic genes (Fig 4), and functional enrichment analysis of these proteins showed they were mainly associated with apoptosis, TGF-beta signaling, adaptive immune system regulation, gene transcription and post-transcriptional protein modification (Fig 5). The gene involved in apoptosis was YWHAQ and the gene involved in TGF-beta signaling was FKBP1A. YWHAQ also joined the process of gene transcription with DDIT4. In adaptive immune system regulation, FKBP1A participates in the calcineurin activation of NFAT and WSB1 and plays a role in antigen processing involving ubiquitination and proteasome degradation. WSB1 is also involved in the post-transcriptional protein modification process, neddylation.  The most over-expressed biomarker in the breast lesion group was DDIT4 (for DNA-damage-inducible transcript 4), also known as REDD1 or RTP801. The major function of the protein encoded by DDIT4 is to inhibit mTORC1, which is induced by various stress stimulus in the hypoxia inducible factor (HIF) family[ 32,33]. Pinto et al reported that high levels of DDIT4 were significantly associated with a worse prognosis (recurrence-free survival, time to progression and overall survival) in several cancer types, including breast cancer [34]. Their previous work indicated that high DDIT4 expression was also an independent factor for a shorter disease-free survival in chemotherapy-resistant triple negative breast tumors [35]. In another report, the dysregulation of basal DDIT4 gene expression in several cancer types (e.g. lung, breast, prostate) can be altered by promyelocytic leukemia (PML) and lead to mTOR activation and cancer progression [36]. DDIT4 also acts as a pro-death transcript in the calcitriol inducing endoplasmic reticulum -stress-like response in breast cancer [37]. Consistent with these reports, in our study DDIT4 was also upregulated in breast lesions, therefore it might serve as a novel prognostic biomarker and is a potential candidate for the development of targeted therapy for breast cancer.  Another upregulated gene, YWHAQ encodes the 14-3-3 proteins, which belong to a group of highly conserved proteins that are essential components of key signaling pathways involved in apoptosis and cell proliferation. These proteins interact with proteins such as Raf, BAD, protein kinase C (PKC), and phosphatidylinositol 3-kinase [38]. The products of YWHAQ (14-3-3ε) regulate TP53 through protein-protein interactions and post-translational modifications [39], and the germline variation in the TP53 network genes PRKAG2, PPP2R2B, CCNG1, PIAS1 and YWHAQ, might affect prognosis and treatment outcome in breast cancer patients [40]. TP53 is closely associated with breast cancer; women who have germline TP53 mutations have a very high risk of breast cancer of up to 85% by age 60 [41]. Combining these reports with our results suggests the TP53 network gene YWHAQ may act as a predictor and new therapy target for breast cancer.  In the present study, FKBP1A participated in both the TGF-beta signaling and calcineurin activation of NFAT. FKBP1A, also named FKBP12, is a member of the FK-506-binding protein (FKBP) family, and its expression in cells is ubiquitous [42, 43]. FKBP1A mediates the immunosuppressive and antitumor effects of rapamycin [44] , widely used in the treatment of breast cancer [45, 46]. One study on Eph receptors and invasive breast carcinoma suggested that the level of FKBP1A was significantly affected by EphB6 ,which was a target mRNA of miR-100, the changes in miRNAs and the target mRNA may have a role in PI3K/Akt/mTOR pathways [47]. FKBP1A has also been shown to inhibit TGF-beta type 1 receptor [48] and it was found overexpressed in childhood astrocytomas, which presented as the EGFR/FKBP12/HIF-2alpha pathway [49]. While an aberration of TGF-beta type 1 receptor is associated with a significantly increased risk of breast cancer [50], FKBP1A may also be associated with an elevated risk of breast cancer, as our study indicated.  Among the downregulated genes, WSB1 is associated with antigen processing, specifically: ubiquitination and proteasome degradation and the post-transcriptional protein modification process, neddylation. WSB-1 (WD-40 repeat-containing SOCS Box protein), is the substrate recognition element of an Elongin Cullin SOCS (ECS box) E3 ubiquitin ligase complex [51] and it was identified as a transcriptional target of HIF [52]. In the only study on the role of WSB1 in breast cancer, Poujade et al evaluated the role of WSB-1 on metastasis promotion both in vitro and in vivo, and they observed that elevated WSB1 expression was associated with decreased distant metastasis-free survival (DMFS) in ER-breast cancer and PR-breast cancer patients, and that WSB-1 knockdown led to decreased metastatic potential in breast cancer hormone receptor-negative models in vitro and in vivo, suggesting WSB-1 may be an important regulator of aggressive metastatic disease in hormone receptor-negative breast cancer [53].  Our results were inconsistent with the above report, however, since WSB1 was decreased in our breast lesion group. The role of WSB1 in other types of cancer is also controversial; this gene was involved in pancreatic cancer progression [54] and metastatic potential of osteosarcoma [52], but its high expression was associated with good prognosis and favorable outcome of neuroblastoma [55]. So the definite function of WSB1 in breast cancer remains unclear. |
| Other information | |  | | |
| Funding | 22 | Give the source of funding and the role of the funders for the present study and, if applicable, for the original study on which the present article is based |  | Huaxia Bangfu Technology Incorporated [http://www.hxjdyl.com/en/gongsijieshao.html] sponsored this research. Changming Cheng, Yali Lyu, Min Wang, Ruirui Zhang are employees of Huaxia Bangfu Technology Inc. Choong-Chin Liew was a consultant of Huaxia Bangfu Technology Inc. The funders had no role in study design, data collection and analysis, decision to publish, or preparation of the manuscript. |

*Give information separately for cases and controls in case-control studies and, if applicable, for exposed and unexposed groups in cohort and cross-sectional studies.

**Note:** An Explanation and Elaboration article discusses each checklist item and gives methodological background and published examples of transparent reporting. The STROBE checklist is best used in conjunction with this article (freely available on the Web sites of PLoS Medicine at http://www.plosmedicine.org/, Annals of Internal Medicine at http://www.annals.org/, and Epidemiology at http://www.epidem.com/). Information on the STROBE Initiative is available at www.strobe-statement.org.
