# Supplementary material for: Peripheral blood transcriptome identifies high-risk benign and malignant breast lesions
Source: PLoS One. 2020 Jun 4;15(6):e0233713. doi: 10.1371/journal.pone.0233713 (PMC7272048; doi:10.1371/journal.pone.0233713)
Supplement: Attachment — Submitted filename: Response to Reviewers - Feb.21.20.docx [file pone.0233713.s005.docx]

Journal requirements:

When submitting your revision, we need you to address these additional requirements.

1. Please ensure that your manuscript meets PLOS ONE's style requirements, including those for file naming. The PLOS ONE style templates can be found at
   http://www.journals.plos.org/plosone/s/file?id=wjVg/PLOSOne_formatting_sample_main_body.pdf and <http://www.journals.plos.org/plosone/s/file?id=ba62/PLOSOne_formatting_sample_title_authors_affiliations.pdf>

**Response: We hope we have met PLOS ONE style requirements.**

2. We noticed you have some minor occurrence(s) of overlapping text with the following previous publication(s), which needs to be addressed:

https://doi.org/10.3892/ol.2018.8577
<https://doi.org/10.1038/s41416-018-0056-3>

In your revision ensure you cite all your sources (including your own works), and quote or rephrase any duplicated text outside the Methods section. Further consideration is dependent on these concerns being addressed.

**Response: the duplicated text with previous publications has been rephrased on page 6/7 and page 20.**

3. Thank you for stating the following in the Financial Disclosure section:

"Funder: Huaxia Bangfu Technology Inc
https://www.hxjdyl.com/en/gongsijieshao
The funders had no role in study design, data collection and analysis, decision to publish, or preparation of the manuscript."
   
We note that one or more of the authors are employed by commercial companies: Huaxia Bangfu Technology Inc and Golden Health Diagnostics Incorporated.

a)    Please provide an amended Funding Statement declaring this commercial affiliation, as well as a statement regarding the Role of Funders in your study. If the funding organization did not play a role in the study design, data collection and analysis, decision to publish, or preparation of the manuscript and only provided financial support in the form of authors' salaries and/or research materials, please review your statements relating to the author contributions, and ensure you have specifically and accurately indicated the role(s) that these authors had in your study. You can update author roles in the Author Contributions section of the online submission form.

Please also include the following statement within your amended Funding Statement.
“The funder provided support in the form of salaries for authors [insert relevant initials], but did not have any additional role in the study design, data collection and analysis, decision to publish, or preparation of the manuscript. The specific roles of these authors are articulated in the ‘author contributions’ section.”
If your commercial affiliation did play a role in your study, please state and explain this role within your updated Funding Statement.

b) Please also provide an updated Competing Interests Statement declaring this commercial affiliation along with any other relevant declarations relating to employment, consultancy, patents, products in development, or marketed products, etc.

Within your Competing Interests Statement, please confirm that this commercial affiliation does not alter your adherence to all PLOS ONE policies on sharing data and materials by including the following statement: "This does not alter our adherence to  PLOS ONE policies on sharing data and materials.” (as detailed online in our guide for authors http://journals.plos.org/plosone/s/competing-interests) . If this adherence statement is not accurate and  there are restrictions on sharing of data and/or materials, please state these. Please note that we cannot proceed with consideration of your article until this information has been declared.

Please include both an updated Funding Statement and Competing Interests Statement in your cover letter. We will change the online submission form on your behalf.

Please know it is PLOS ONE policy for corresponding authors to declare, on behalf of all authors, all potential competing interests for the purposes of transparency. PLOS defines a competing interest as anything that interferes with, or could reasonably be perceived as interfering with, the full and objective presentation, peer review, editorial decision-making, or publication of research or non-research articles submitted to one of the journals. Competing interests can be financial or non-financial, professional, or personal. Competing interests can arise in relationship to an organization or another person. Please follow this link to our website for more details on competing interests: http://journals.plos.org/plosone/s/competing-interests

**Response: We have added the following statements to our Cover Letter:**

**Funding Statement**

**Huaxia Bangfu Technology Incorporated [http://www.hxjdyl.com/en/gongsijieshao.html] sponsored this research. Changming Cheng, Yali Lyu, Min Wang, Ruirui Zhang are employees of Huaxia Bangfu Technology Inc. Choong-Chin Liew was a consultant of Huaxia Bangfu Technology Inc. The funder provided support in the form of salaries for authors [C. Cheng, Y. Lyu, M. Wang, R. Zhang], but did not have any additional role in the study design, data collection and analysis, decision to publish, or preparation of the manuscript. The specific roles of these authors are articulated in the ‘author contributions’ section.**

**Competing Interests Statement**

**The authors have read the journal’s policy and have the following conflicts: Changming Cheng, Yali Lyu, Min Wang, Ruirui Zhang are employees of Huaxia Bangfu Technology Inc who sponsored this research. Choong-Chin Liew was a consultant of Huaxia Bangfu Technology Inc and the founder of Golden Health Diagnostics Incorporated. None of the other authors has any competing interests. There are no patents, products in development or marketed products to declare. This does not alter the authors’ adherence to all the PLOS ONE policies on sharing data and materials.**

**Author contributions**

| **Hong Hou** | **Conceptualization, Data curation, Resources** |
| --- | --- |
| **Yali Lyu** | **Conceptualization, Project administration, Writing - original draft,** |
| **Jing Jiang** | **Data curation, Resources** |
| **Min Wang** | **Data curation, Formal analysis, Software** |
| **Ruirui Zhang** | **Formal analysis, Methodology, Visualization** |
| **Changming Cheng** | **Conceptualization, Supervision, Writing - review & editing, Funding acquisition** |
| **Choong-Chin Liew** | **Conceptualization, Writing - review & editing** |
| **Binggao Wang** | **Conceptualization, Data curation, Resources, Supervision** |

[Note: HTML markup is below. Please do not edit.]

Reviewers' comments:

Reviewer's Responses to Questions

**Comments to the Author**

1. Is the manuscript technically sound, and do the data support the conclusions?

The manuscript must describe a technically sound piece of scientific research with data that supports the conclusions. Experiments must have been conducted rigorously, with appropriate controls, replication, and sample sizes. The conclusions must be drawn appropriately based on the data presented. 

Reviewer #1: Partly

Reviewer #2: Yes

Reviewer #3: No

2. Has the statistical analysis been performed appropriately and rigorously? 

Reviewer #1: Yes

Reviewer #2: Yes

Reviewer #3: No

3. Have the authors made all data underlying the findings in their manuscript fully available?

The [PLOS Data policy](http://www.plosone.org/static/policies.action#sharing) requires authors to make all data underlying the findings described in their manuscript fully available without restriction, with rare exception (please refer to the Data Availability Statement in the manuscript PDF file). The data should be provided as part of the manuscript or its supporting information, or deposited to a public repository. For example, in addition to summary statistics, the data points behind means, medians and variance measures should be available. If there are restrictions on publicly sharing data—e.g. participant privacy or use of data from a third party—those must be specified.

**Data Availability Statement: All relevant data are within the manuscript and its supporting information. The gene expression profiles and the risk score calculated by predictive model based on 10-gene panel were detailed listed in S1 and S2 Tables of Support Information.**

Reviewer #1: No

Reviewer #2: Yes

Reviewer #3: Yes

4. Is the manuscript presented in an intelligible fashion and written in standard English?

PLOS ONE does not copyedit accepted manuscripts, so the language in submitted articles must be clear, correct, and unambiguous. Any typographical or grammatical errors should be corrected at revision, so please note any specific errors here.

Reviewer #1: Yes

Reviewer #2: Yes

Reviewer #3: Yes

5. Review Comments to the Author

Please use the space provided to explain your answers to the questions above. You may also include additional comments for the author, including concerns about dual publication, research ethics, or publication ethics. (Please upload your review as an attachment if it exceeds 20,000 characters)

Reviewer #1: In this manuscript authors have reported a method for detection of breast lesions using peripheral blood transcriptomic profiling. Overall the manuscript has been designed well but some issues need to be addressed. Minor: The font in Figure 1 should be made clearer. It was a little hard to read.

**Response: Figure 1 was rephrased on page 13 and I hope that the font is clearer now.**

**Fig. 1. Heat map of gene expression and hierarchical cluster diagram showing 10 single candidate genes (A) and a 10-gene combination (B) for clustering the 151 samples including 107 breast lesions and 44 normal controls. Dendrogram generated using ‘‘Heatmap’’ function in R with default settings.**

Major: Definitely a larger sample size and more validation studies will be needed to support this method. Did authors try RNAseq instead of microarray? They need to address the fact that microarray has a limitation in which it does not allow accurate assessment of low signal intensities. And may also give background hybridization. Also the authors need to provide information on the ER, HER2 status of the lesions if available.

**Response: Thanks for this constructive suggestion. Certainly RNAseq is a new and powerful tool for transcriptomic study, especially suitable for exploring unknown transcript fragments and RNA sequence variation. However, Affymetrix microarray has also been proven to be a robust and accurate technology for gene expression profiling studies. The goal of our study is to develop a technology that can exploit gene expression signatures characteristic of various breast lesion types. In previous publications, we identified a series of gene biomarkers for cancer (see references 17,31,32) that were characterized using microarray analysis and were further confirmed by RT-PCR. Thus we think Affymetrix microarray analysis is a reliable method to study gene expression signatures, as in this study.**

**We have added to the manuscript, on page 22:**

**“RNA sequencing (RNAseq) has been proven an efficient tool for transcriptome analysis, especially for exploring expression signatures of unknown transcript fragments and revealing the signaling pathways beneath. An interesting subject for future study would be to compare the variations in gene expression signatures between RNAseq and the microarray method.”**

**The treatment of data for Affymetrix microarray hybridization has been described in detail in our previous report (Chao S, Liew CC. (2015). "Mining the Dynamic Genome: A Method for Identifying Multiple Disease Signatures Using Quantitative RNA Expression Analysis of a Single Blood Sample." Microarrays 4: 671-689.). We have added this as a reference (#23) in Materials and Methods section on page 7.**

**Here for your reviewers’ information is a link to this article: [https://www.ncbi.nlm.nih.gov/pmc/articles/PMC4996407/]**

**ER and HER2 information from patients was not completed and was unavailable, as only some patients received ER/HER2 examination in this study.**

Reviewer #2: The authors have described a ten-gene panel signature that can be used as a predictive model to detect high risk breast lesions as well as breast cancer. The authors have performed the research very comprehensively and all the analyses performed is scientifically sound. While I do believe the research performed is of significant importance in the field, I do request the authors to address a few concerns before the manuscript can be accepted for publication:

1) On page 9, the authors mention that patients with breast cancer were older than the controls as well as the ones with high-risk lesions. How do the authors know that the ten gene panel signature isn't simply reflective of aging and is directly related to the process of carcinogenesis.

**Response: Certainly a larger sample size and more validation studies will be needed to support the findings described in our study. In a future report, we would like to enroll more healthy controls, more women with high risk breast lesions and more breast cancer patients with better-matched age distributions.**

**We have therefore added on pp. 21/22:**

**“…our samples show some age variation among the healthy controls, the women with high risk lesions and the breast cancer patients. Although we have shown in our discussion that the gene biomarkers we identified are related more to the process of carcinogenesis than to aging, we intend to confirm the effectiveness of our data mining method in further studies, using a larger sample size with age-matched patients.**

2) Did the author check for the known drivers of cancer and in particular breast cancer such as BRCA1,BRCA2, p53 and such other genes? Or was the analysis done in a way to find out the other genes beyond the ones that were already identified in the literature?

**Response: This is an interesting question for further study. The mutations of BRCA1, BRCA2 and p53 genes have been widely observed in breast cancer tissue cells and are expected to play important roles in carcinogenesis. However in the present study, we sought to identify an altered expression signature as a characteristic of blood cells rather than of cancer tissue cells. We thus did not expect to find correlations in gene expression alterations between blood cells and breast cancer cells. By analyzing the gene expression profiles of peripheral blood cells, we found that those driver genes of cancer cells such as BRCA1, BRCA2, p53 and other genes did not exhibit significant variation in blood cells as compared to healthy controls （added on Page 21）. However, the gene biomarkers we identified in the blood-based transcriptome are shown to be related to the process of carcinogenesis in our bio-information analysis, as discussed in detail in Discussion section on pages 18-21. Further study to identify the signaling pathways of blood cells and their interaction with cancer cells is warranted to better understand the roles of blood cells in the carcinogenesis process.**

**We have added to our manuscript on p. 21:**

**“Mutations and abnormalities in the expression of *BRCA1, BRCA2* and *p53* genes have been widely observed in breast cancer tissue cells and are thought to play important roles as driver genes in carcinogenesis. In our study, however, *BRCA1, BRCA2*, *p53* and other driver genes did not exhibit significant variations in expression between breast lesions and healthy controls in peripheral blood cells. This difference might be attributed to differences in biological functions between blood cells and tissue cells in the process of carcinogenesis. Further study is required in order to identify the signaling pathways of blood cells and their interaction with cancer cells to better understand the roles of blood cells in carcinogenesis.”**

3) Is there a way to follow up to see if the 3 'false-positive' normal samples in their test set go on to develop high risk breast lesions and therefore check if the ten-gene panel signature is actually predictive of the early stage carcinogenesis/development of high-risk lesions?

**Response: We have added on p.17:**

**“We are planning to follow over the next 3-5 years those healthy controls who presented with false-positive results, in order to determine whether some of them will go on to develop high-risk lesions or breast cancer.”**

4) While the premise of the project and that of the manuscript is to discover a ten-gene signature panel that is predictive of developing high risk breast lesions, all the samples analyzed are grade 3 or 4 (BI-RADS). Can authors actually test if this panel is actually predictive of development of high-risk lesions by testing their gene signature panel in the grade I or grade II patients?

**Response: Due to the limited sample size in the present study, the number of grade I or grade II patients is too small to generate a reliable predictive result. In a future study, we plan to recruit more patients with grade I or grade II to further validate the performance of this ten-gene panel in early detection of high-risk lesions and breast cancer.**

5) Have the authors tested to see if among the 147 protein interacting partners, the transcripts of the interacting partners were also altered in their RNAseq analyses? This is only to understand if multiple genes in the pathways described in the manuscript have been altered, and therefore narrow down a potential pathway of interest for further studies?

**Response: Thanks for this constructive suggestion. In the present study, we aim to identify blood-based genomic signatures that discriminate high-risk lesion and breast cancer from healthy controls using microarray analysis. As the reviewer suggests, RNAseq is a new and powerful tool for transcriptomic study, and is especially suitable for identifying unknown transcript fragments and exploring their signaling pathways. We would like to use RNAseq method to analyze and confirm potential pathways of interesting blood transcripts in a future study.**

**We have added to the manuscript, on page 22:**

**“RNA sequencing (RNAseq) has been proven an efficient tool for transcriptome analysis, especially for exploring expression signatures of unknown transcript fragments and revealing the signaling pathways beneath. An interesting subject for future study would be to compare the variations in gene expression signatures between RNAseq and the microarray method.”**

Once these comments are addressed, the manuscript can be considered for publication.

Reviewer #3: Comments:
1. It is not clear from the description about the parameters used to identify the 10 candidate genes.

**Response: the data mining method for blood-based genomic signatures has been described in our previous reports ([17,31,32]). To clarify this in the current manuscript, we have added a new reference to a previous publication that has described in detail the data mining process for blood-based signature identification (Samuel Chao, C. C., Choong-Chin Liew (2015). "Mining the Dynamic Genome: A Method for Identifying Multiple Disease Signatures Using Quantitative RNA Expression Analysis of a Single Blood Sample." Microarrays 4: 671-689.). This is added in Materials and Methods section on page 8.**

**We hope that the reviewer will find our methods to be robust and well-validated. Here for the reviewer’s information is a link to this article:** [**https://www.ncbi.nlm.nih.gov/pmc/articles/PMC4996407/]**

2. It would be interesting to know the comparison of the various gene expression profile between the high risk benign breast lesion vs malignant breast lesions

**Response: This is an interesting suggestion for further study. In the present study, we expected to develop blood-based genomic signatures to discriminate high-risk lesions and breast cancer from healthy controls. In a future study, we would like to explore the evolution from high risk benign lesion to malignant breast carcinoma by comparing variations between high risk benign breast lesion and malignant breast lesions.**

3. Since the sample size is small it is important to use an age matched control.

**Response: we have added our response to page 22:**

**“…our samples show some age variation among the healthy controls, the women with high risk lesions and the breast cancer patients. Although we have shown in our discussion that the gene biomarkers we identified are related more to the process of carcinogenesis than to aging, we intend to confirm the effectiveness of our data mining method in further studies, using a larger sample size with age-matched patients.”**

4. The false positivity observed in 3 of the 19 controls of the predictive model is not justified despite of small sample size

**Response: We have added to page 17:**

**“We are planning to follow those healthy controls who presented with false-positive results over the next 3-5 years, in order to determine whether some of them will go on to develop high-risk lesions or breast cancer.”**

5. Significant figures should be considered while calculating Fold change.

**Response: The fold changes of the interesting 10 genes are listed in Table 3. Variation in blood gene expression profiles between cancer samples and heathy samples is usually not as significant as variations found between cancer cells and surrounding healthy tissue cells. This issue makes blood-based biomarker screening more difficult. To overcome these challenges we have developed an effective strategy for identifying blood based genomic signatures as referenced above in Question 1:** [**https://www.ncbi.nlm.nih.gov/pmc/articles/PMC4996407/**](https://www.ncbi.nlm.nih.gov/pmc/articles/PMC4996407/)

| **Table 3 Candidate biomarkers for distinguishing breast lesions from controls** | | | | |
| --- | --- | --- | --- | --- |
| **Probe set ID** | **Gene Symbol** | **Gene Title** | **Fold Change** | **Regulation** |
| 202887_s_at | *DDIT4* | DNA damage inducible transcript 4 | 2.0014469 | up |
| 214953_s_at | *APP* | amyloid beta (A4) precursor protein | 1.97339 | up |
| 214119_s_at | *FKBP1A* | FK506 binding protein 1A | 1.8358978 | up |
| 202876_s_at | *PBX2* | pre-B-cell leukemia homeobox 2 | 1.7287292 | up |
| 200693_at | *YWHAQ* | tyrosine 3-monooxygenase/ tryptophan 5-monooxygenase activation protein, theta | 1.0801506 | up |
| 217317_s_at | *HERC2P2* | hect domain and RLD 2 pseudogene 2 | -1.2864129 | down |
| 208835_s_at | *LUC7L3* | LUC7-like 3 pre-mRNA splicing factor | -1.3374902 | down |
| 201296_s_at | *WSB1* | WD repeat and SOCS box containing 1 | -1.350631 | down |
| 201101_s_at | *BCLAF1* | BCL2-associated transcription factor 1 | -1.4449192 | down |
| 1554178_a_at | *FAM126B* | family with sequence similarity 126, member B | -1.4458523 | *down* |

6. PLOS authors have the option to publish the peer review history of their article ([what does this mean?](https://journals.plos.org/plosone/s/editorial-and-peer-review-process#loc-peer-review-history)). If published, this will include your full peer review and any attached files.


If you choose “no”, your identity will remain anonymous but your review may still be made public.


**Do you want your identity to be public for this peer review?** For information about this choice, including consent withdrawal, please see our [Privacy Policy](https://www.plos.org/privacy-policy).

Reviewer #1: No

Reviewer #2: No

Reviewer #3: No


[NOTE: If reviewer comments were submitted as an attachment file, they will be attached to this email and accessible via the submission site. Please log into your account, locate the manuscript record, and check for the action link "View Attachments". If this link does not appear, there are no attachment files to be viewed.]


While revising your submission, please upload your figure files to the Preflight Analysis and Conversion Engine (PACE) digital diagnostic tool,<https://pacev2.apexcovantage.com/>. PACE helps ensure that figures meet PLOS requirements. To use PACE, you must first register as a user. Registration is free. Then, login and navigate to the UPLOAD tab, where you will find detailed instructions on how to use the tool. If you encounter any issues or have any questions when using PACE, please email us at [figures@plos.org](mailto:figures@plos.org). Please note that Supporting Information files do not need this step.
