# Supplementary material for: Peripheral blood transcriptome identifies high-risk benign and malignant breast lesions
Source: PLoS One. 2020 Jun 4;15(6):e0233713. doi: 10.1371/journal.pone.0233713 (PMC7272048; doi:10.1371/journal.pone.0233713)
Supplement: Attachment — Submitted filename: Response to Reviewers_April.05.20.docx [file pone.0233713.s006.docx]

Response to Reviewers

Reviewers' comments:
Reviewer's Responses to Questions
**Comments to the Author**
1. If the authors have adequately addressed your comments raised in a previous round of review and you feel that this manuscript is now acceptable for publication, you may indicate that here to bypass the “Comments to the Author” section, enter your conflict of interest statement in the “Confidential to Editor” section, and submit your "Accept" recommendation.

Reviewer #1: All comments have been addressed

Reviewer #2: All comments have been addressed

Reviewer #3: All comments have been addressed

2. Is the manuscript technically sound, and do the data support the conclusions?

The manuscript must describe a technically sound piece of scientific research with data that supports the conclusions. Experiments must have been conducted rigorously, with appropriate controls, replication, and sample sizes. The conclusions must be drawn appropriately based on the data presented. 

Reviewer #1: (No Response)

Reviewer #2: Partly

Reviewer #3: Yes

3. Has the statistical analysis been performed appropriately and rigorously? 

Reviewer #1: (No Response)

Reviewer #2: Yes

Reviewer #3: Yes

4. Have the authors made all data underlying the findings in their manuscript fully available?

The [PLOS Data policy](http://www.plosone.org/static/policies.action#sharing) requires authors to make all data underlying the findings described in their manuscript fully available without restriction, with rare exception (please refer to the Data Availability Statement in the manuscript PDF file). The data should be provided as part of the manuscript or its supporting information, or deposited to a public repository. For example, in addition to summary statistics, the data points behind means, medians and variance measures should be available. If there are restrictions on publicly sharing data—e.g. participant privacy or use of data from a third party—those must be specified.

Reviewer #1: (No Response)

Reviewer #2: Yes

Reviewer #3: Yes

5. Is the manuscript presented in an intelligible fashion and written in standard English?

PLOS ONE does not copyedit accepted manuscripts, so the language in submitted articles must be clear, correct, and unambiguous. Any typographical or grammatical errors should be corrected at revision, so please note any specific errors here.

Reviewer #1: (No Response)

Reviewer #2: Yes

Reviewer #3: Yes

6. Review Comments to the Author

Please use the space provided to explain your answers to the questions above. You may also include additional comments for the author, including concerns about dual publication, research ethics, or publication ethics. (Please upload your review as an attachment if it exceeds 20,000 characters)

Reviewer #1: (No Response)

Reviewer #2: I believe while the authors have addressed all the comments, some of them have been loosely addressed. While most of the ones are fairly minor, there are three comments that still concern me which certainly affect the validity of the studies.

1) The samples in the two groups are not age matched. In response to this comment, the author's have added this in text of the manuscript '“…our samples show some age variation among the healthy controls, the women with high risk lesions and the breast cancer patients. Although we have shown in our discussion that the gene biomarkers we identified are related more to the process of carcinogenesis than to aging, we intend to confirm the effectiveness of our data mining method in further studies, using a larger sample size with age-matched patients'. I believe this as a bit of ''hand-waving'' by saying that the genes biomarkers are more related to carcinogenesis than to aging. In my opinion, while they are related to carcinogenesis, they could still be related to aging and if so the predictive value of these genes would be fairly limited as with a larger sample size they may also see a significant increase in false positives.

**Response: Thanks for the reviewer’s valuable comments. Age has been regarded as an important risk factor for cancer, as the incidence of most cancers increases with age. In this study, which is restricted by a limited sample size, we tried to optimize the algorithm to eliminate the interference of age factors as much as possible. However, as the reviewer mentioned, it is hard to confirm that the biomarkers derived are completely unrelated to age. We also state this as a limitation in the Discussion section on page 22. In further study, we plan to validate the biomarkers and the algorithm using a larger sample size with age-matched patients.**

2) In their analysis, they had 3 normal samples show up as a false-positive. While the authors state that they plan to follow this patient for 3-5 years, there is no way to ensure this would happen for sure and therefore not predictive of early stage carcinogenesis and breast lesions as the authors state.

**Response: We would like to confirm whether those 3 false positive samples are true negative samples by following them up in the next few years. For us, the purpose of this study is to develop a blood test to predict early stage carcinoma and breast lesions. Thus, it would be interesting and valuable to our future research to confirm whether the biomarkers identified from this retrospective study are in fact effective in predicting early stage breast lesions. We expect to explore this in prospective studies of breast lesions in the future. We also state this aim in our Discussion section on Page 17.**

3) It is still surprising to me that known drivers of carcinogenesis such as p53, BRCA1, BRCA2 does not show up in their study. While authors state 'This difference might be attributed to differences in biological functions between blood cells and tissue cells in the process of carcinogenesis.', I disagree with this argument. The assumption behind assessing peripheral gene signature is that blood contains tumor cells that are now in circulation after metastasis from the primary tumor. With this rationale, some of the known driver's should have been detected.

**Response: The gene mutations related to carcinogenesis, such as p53, BRCA1 / BRCA2, have been widely observed in breast tumor cells; however they have not been identified in our study. There are several possible reasons for this. Although tumor cells could be released into a patient’s peripheral blood, the proportion of such cells as compared with white blood cells would be very low, even for patients with advanced disease. White blood cells predominate in the cell spectrum of peripheral blood, and therefore blood gene expression signatures would largely reflect these abundant blood white cells rather than the rare circulating tumor cells. In addition, as blood white cells and tumor cells play different biological roles in the process of carcinogenesis their gene expression profiles also differ. Gene expression variations in blood white cells, for example, more likely reflect interactions between the immune system and the tumor rather than reflecting intrinsic changes within the tumor cells themselves. These differences might be an important reason why the driver genes that have been observed in tumor cells did not show abnormal signals in the gene expression profile of peripheral blood in this study.**

**We have added this in the Discussion section on Pages 21-22.**

Reviewer #3: The authors have carefully addressed the comments to the reviewers. They have also added additional references to describe the methods used in the study . However, some comments can be addressed only by further study and using bigger sample size.
I believe the authors would consider the comments suggested by the reviewers and address them in their future studies.

**Response: on behalf of all authors, we appreciate the reviewers’ valuable suggestions very much and would like to consider them seriously in our future studies.**

7. PLOS authors have the option to publish the peer review history of their article ([what does this mean?](https://journals.plos.org/plosone/s/editorial-and-peer-review-process#loc-peer-review-history)). If published, this will include your full peer review and any attached files.


If you choose “no”, your identity will remain anonymous but your review may still be made public.


**Do you want your identity to be public for this peer review?** For information about this choice, including consent withdrawal, please see our [Privacy Policy](https://www.plos.org/privacy-policy).

Reviewer #1: No

Reviewer #2: No

Reviewer #3: No


[NOTE: If reviewer comments were submitted as an attachment file, they will be attached to this email and accessible via the submission site. Please log into your account, locate the manuscript record, and check for the action link "View Attachments". If this link does not appear, there are no attachment files to be viewed.]
